# The Effect of Ingroup vs. Outgroup Members' Behavior on Charity Preference: A Drift-Diffusion Model Approach

**DOI:** 10.3389/fpsyg.2022.854747

**Published:** 2022-05-27

**Authors:** Lars M. Reich, Luisa A. M. Mahr, Martina Vacondio, Afreen S. Khalid

**Affiliations:** ^1^Department of Digital Age Research Center, University of Klagenfurt, Klagenfurt, Austria; ^2^Department of Social Psychology, University of Klagenfurt, Klagenfurt, Austria; ^3^Department of Cognitive Psychology, University of Klagenfurt, Klagenfurt, Austria

**Keywords:** charitable donations, ingroup, outgroup, DDM, social information, conformity

## Abstract

Providing potential donors with information about the behavior of others (i.e., social information) is an increasingly used strategy to nudge prosocial decision-making. In the present study, we investigated the effect of ingroup vs. outgroup information on participants' charity preferences by applying a Drift Diffusion Model (DDM) approach. In a joint evaluation scenario, we manipulated different levels of ingroup/outgroup preference ratios for two charities within subjects. Every subject was presented with three stimulus types (i.e., high, medium, and low ingroup ratio) randomized in 294 trials divided into six blocks. We expected that for stimuli with a high ingroup/outgroup ratio, participants should more often and faster decide for the ingroup's most favored charity. We expected that the speed of evidence accumulation will be higher the larger the ingroup/outgroup ratio. Additionally, we investigated whether variations in model parameters can explain individual differences in participants' behaviors. Our results showed that people generally followed ingroup members' preferences when deciding for a charity. However, on finding an unexpected pattern in our results, we conducted *post-hoc* analyses which revealed two different behavioral strategies used by participants. Based on participants' decisions, we classified them into “equality driven” individuals who preferred stimuli with the least difference between ingroup and outgroup percentages or “ingroup driven” individuals who favored stimuli with the highest ingroup/outgroup ratio. Results are discussed in line with relevant literature, and implications for practitioners are given.

## 1. Introduction

Donations made by private persons make up a large part of charitable giving. In the UK alone, there are more than 200,000 registered charity organizations, making it important to answer not only what motivates people to donate in general but also what motivates them to select a specific cause, organization, or program to donate to.

Research has identified various driving factors in charitable giving decisions, for example, the neediness of the recipient (Kogut and Ritov, 2005), identifiability of the donor (Small et al., 2007; Lee and Feeley, 2016), or personality characteristics of the donor such as social value orientation (Van Lange et al., 2007).

An increasingly used and promising strategy of donor acquisition is to provide potential donors with information about the behavior of others (e.g., amount given by previous donors), that is, to implement social information (van Teunenbroek et al., 2020). Learning about others' behavior establishes a social norm to which people are generally inclined to adapt (e.g., Festinger, 1954; Bernheim, 1994). Conformity can foster one's social acceptance, and others can serve as a source of information on what is more effective to do in a given situation (Cialdini and Goldstein, 2004), especially if a situation is new, ambiguous, or uncertain (Goldstein et al., 2008). Based on this knowledge, various programs and campaigns have implemented a social norm approach to promote desirable behaviors (Schultz et al., 2007), including charitable giving (Minguez and Sese, 2021). However, evidence on the effect of social information on donations is not as consistent as one might assume, with several studies showing a positive effect while others find no or even negative effects (van Teunenbroek et al., 2020). Therefore, it is crucial to identify contextual factors under which social information is particularly effective in promoting charitable giving.

A relevant contextual factor is the source of social information, i.e., whose previous behavior is provided to potential donors (van Teunenbroek et al., 2020). According to social identity theory, people base their self-concept in part on the social groups they belong to, accompanied by a differentiation of the social world into in- and outgroups (Tajfel et al., 1979; Turner, 1999). Besides a general tendency to favor ingroups over outgroups (Aberson et al., 2000), research has demonstrated that people are generally more receptive to social influence from ingroup rather than outgroup members (e.g., Abrams and Hogg, 1990; Knippenberg and Wilke, 1992). Moreover, studies on the influence of norms on prosocial behavior show the superiority of ingroup-specific over general norms (e.g., Lede et al., 2019).

Although research in the donation domain has frequently investigated the role of group membership in victims or recipients (e.g., favoring ingroup victims; James and Zagefka, 2017), surprisingly, the group membership of other donors has received little attention. One study examined the effect of ingroup vs. outgroup average donations' anchors on the decision to donate and the amount donated (Hysenbelli et al., 2013). They demonstrated that people tend to donate more when high anchors are attributed to ingroup donors than outgroup donors in a separate evaluation setting. However, to the best of our knowledge, no studies to date have examined the effect of ingroup vs. outgroup information in a joint evaluation scenario, leaving it unclear how group membership affects donation decisions when in- and outgroup information is presented simultaneously. Former research has emphasized that both the decision-making process and its outcomes may differ if attributes and alternatives are evaluated relative to rather than isolated from each other (e.g., Payne et al., 1993; Caviola et al., 2014). Moreover, when the information provided refers to in- and outgroup members' behavior, a joint evaluation scenario creates an intergroup context, increasing the salience of social identity and social intergroup comparison (e.g., Turner, 1999). Therefore, the first aim of our study was to test the effect of ingroup vs. outgroup information on charity preference in a joint evaluation scenario. Further, by applying the Drift-Diffusion Model (DDM, Ratcliff, 1978; Ratcliff and McKoon, 2008; Ratcliff et al., 2016), our second goal was to understand the process of how ingroup vs. outgroup information influences charity preferences while accounting for interindividual differences.

The DDM is a computational model and describes decision-making. Applying the model assumes that evidence is accumulated over time until an evidence threshold is reached that triggers the decision. We assume that decision-makers extract evidence from provided information and receive it from memory. The accumulation process, called drift rate (*v*), tends in a stochastic manner to either the in- or outgroup response, depending on the evidence. The larger the value of the drift rate, the higher the accuracy and the faster the response (Lerche and Voss, 2019). Three additional main parameters are threshold (*a*), non-decision time (*t*_0_), and starting point (*z*). The threshold *a* defines the relative distance between the thresholds for both choice options. The larger the *a*, the more information needs to be accumulated. In a speed-accuracy manipulation, it has been shown that by deciding as accurately as possible, the threshold *a* increases, as does the caution and the accuracy of the decision-maker (Ratcliff and Rouder, 1998). The non-decision time summarizes processes that are not directly involved (e.g., motor responses) in the decision process. When forcing participants to press a key three times in a row (instead of just once) to respond, the non-decision time *t*_0_ increases (Lerche and Voss, 2019). The starting point bias *z* indicates whether participants are biased toward a response before seeing the task. By randomizing the trial order, the relative bias *z* should be at 0.50, which means participants are unbiased. Three variability parameter (i.e., *sz*, *st*_0_, *sv*) ensure the intertrial variability (for more information; e.g., Voss et al., 2004, 2013; Ratcliff and McKoon, 2008; Wagenmakers, 2009).

Specifically, by applying the DDM, we investigated how varying proportions of in- vs. outgroup members' decisions influence individual decision-making. We expected that for stimuli with a high ingroup ratio, participants should more often and faster decide for the ingroup's most favored charity and that we will find this phenomenon in the drift rate of the model. Furthermore, we assumed that individuals with stronger ingroup identification show even stronger effects on the drift rate parameter. Thus, individuals with a high sense of ingroup-identification have higher drift rate values for charities favored by the ingroup. Nevertheless, it is common in behavioral science that the observed effects do not affect participants homogeneously. Therefore, we also investigated whether variations in model parameters can explain individual differences in participants' behaviors which tend to remain undetected in the analysis of aggregated data (e.g., negligible or even reverse effects).

## 2. Materials and Methods

### 2.1. Participants and Design

Based on the literature that used a DDM approach similar to our study, we planned for a sample size of at least *N* = 30 participants (see, e.g., Krajbich et al., 2012). To compensate for potential dropouts, we increased the study's sample size to *N* = 39 (16 women, 22 men, 1 diverse, *M*_*age*_ = 30.44, *SD* = 9.90). Participants needed to be native English speakers and at least 18 years old to qualify for the study. We recruited UK citizens *via* the online subjects' pool Prolific. Prolific holds good recruitment standards and explicitly informs participants that they are recruited for participation in research (Palan and Schitter, 2018). For the duration of our study (i.e., 1 h), participants received £7.50. No participants were excluded from the analysis. To ensure data quality, we applied the same outlier handling (Lerche and Voss, 2019) did (see Section 2.4).

To examine the effects of other donors' group membership on individual donation decisions, we created an online experiment with lab.js (Henninger et al., 2021). We manipulated participants' group membership experimentally in the first step, manipulated different levels of ingroup/outgroup preference for the two charities within subjects in the second step, and measured participants' decisions as well as decision times. We further measured ingroup identification to evaluate whether ingroup compliance might be elevated for those showing stronger ingroup identification. The study was reviewed and approved by the University's internal ethics committee before data collection.

### 2.2. Procedure

After obtaining informed consent, the minimal group priming was applied to manipulate participants' group membership (Tajfel, 1970; Bornstein et al., 1983). Across eight trials, participants had to choose which of two presented paintings (either painted by Klee or Kandinsky) they liked the most. Subsequently, all participants received false feedback that Kandinsky painted most of the pictures they preferred and that they would thus be assigned to the “Kandinsky group”. Next, they completed a manipulation check, indicating their feelings (sympathetic, warm, soft-hearted, compassionate, tender, moved; Batson et al., 1997) toward the Kandinsky- and Klee group on a 7-point Likert scale (1 = “not at all” to 7 = “very much”). A paired sample *t*-test indicated our manipulation was successful: participants had significantly more empathy toward their ingroup (Kandinsky; *M* = 3.47, *SD* = 1.45) than the outgroup (Klee; *M* = 2.91, *SD* = 1.40), *t*_(38)_ = 4.50, *p* < 0.001, *d* = 0.72, 95% CI = [0.31, 0.81]. After this, participants answered four items on the strength of identification with their ingroup (e.g., “I see myself as a Kandinsky member”; Doosje et al., 1995) on a 7-point-Likert-scale (1 = “not at all” to 7 = “very much”).

In the main experiment, participants were asked to make several decisions on which of two charities they preferred to donate to. For every decision, participants were provided with information on the in- and outgroup members' ostensible behavior (i.e., the number of people from the in- and outgroup that decided to donate to the two charities), resulting in a 2x2 table. Across trials, different levels of in- vs. outgroup preferences were manipulated within subjects by implementing different stimulus types (see stimuli description). The trials' values in columns (A, B) were summed to 100 to ensure comparable choice options regarding the number of donors. They indicated how many donors had already donated to that charity option. The rows (Kandinsky, Klee) are independent and provided information on how many group members donated to one of the charity options.

**Figure 1:**
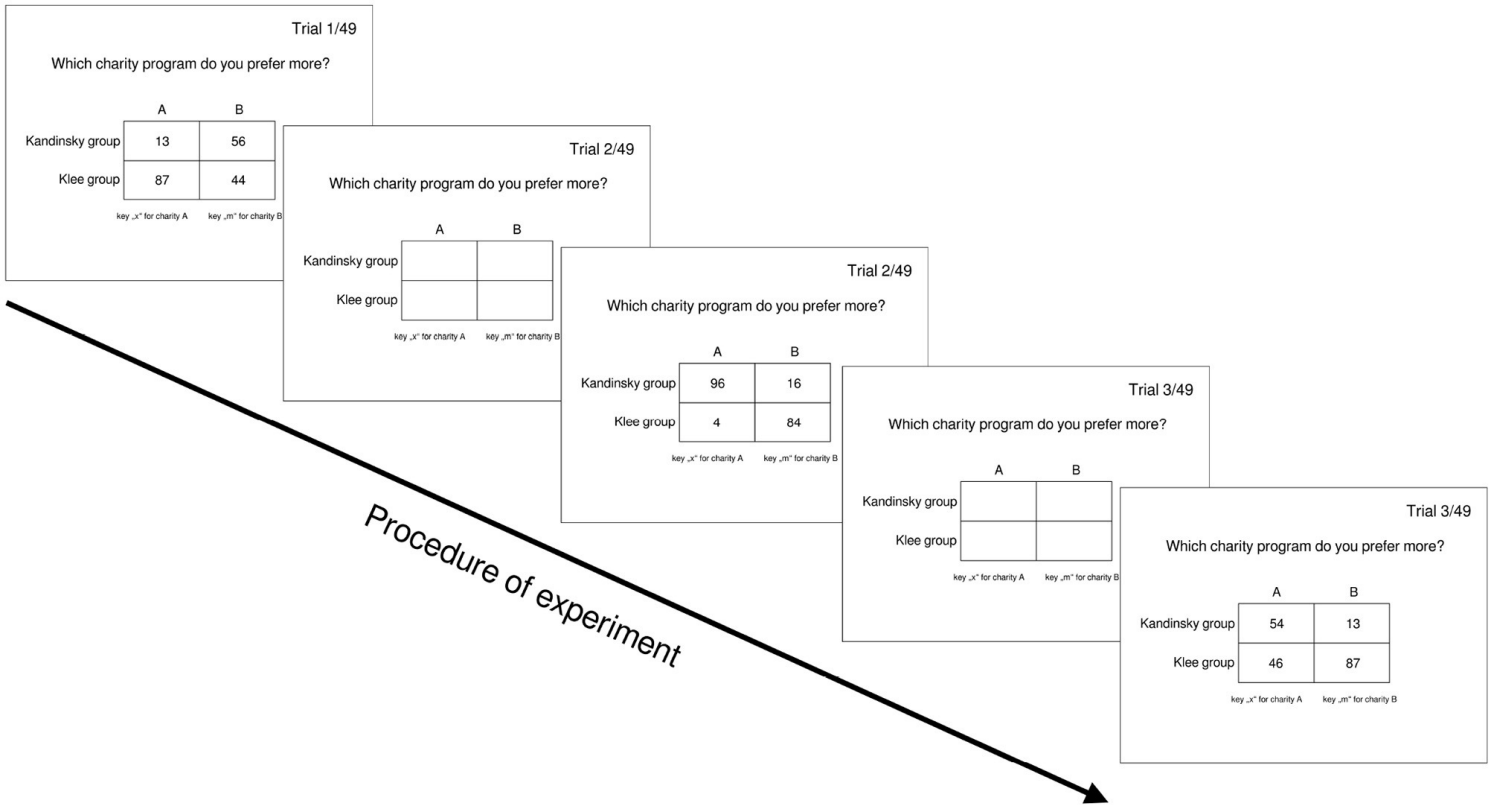
Exemplary representation of the experimental procedure. Between trials, a blank screen was displayed for 200, 400, or 600 ms.

After eight practice trials, every subject was presented with 294 trials divided into six blocks, with 49 trials each. Every trial started randomly after 200, 400, or 600 ms, after responding by either pressing the key “x” for option A or “m” for option B (see Figure 1). Participants had a response window of 5 s, which, when reached, automatically triggered the next trial. After each block, participants had the opportunity to take a break. The blocks were randomized across participants and differed in the instruction that the following 49 decision tasks were charities for either: “cancer charities”, “disabled charities”, “poverty charities”, “medical charities”, “elderly charities”, or “children's health charities”. The general design of this experiment was similar to experimental procedures from multi-attribute decision experiments (e.g., Trueblood, 2012).

In the last part of the study, participants answered demographic questions (age, gender, weekly income) and were given the possibility to describe their decision strategy within an open-response format before being fully debriefed.

### 2.3. The Stimuli

**Figure 2:**
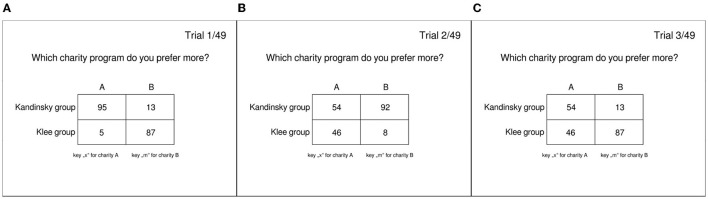
Example of the three stimulus types. Left **(A)** stimulus type 1 (low difficulty), middle **(B)** stimulus type 2 (medium difficulty), right **(C)** stimulus type 3 (high difficulty).

We created three stimulus types (see Figure 2), reflecting three different difficulty levels to follow the ingroup: low vs. medium vs. high. For each stimulus type, we constructed 49 different stimuli by varying the ingroup preference, e.g., stimulus type 1 for option A between [92, 98] and for option B between [12, 18], in a way that each preference for option A is combined with each preference of option B. Each of the 49 stimuli was presented twice by presenting option A, either left or right (and vice versa for option B). The same procedure was applied for stimulus type 2 (option A [92,98], option B [52,58]), and stimulus type 3 (option A [52,58], option B [12,18]) resulting in 294 different stimuli that were randomized for each subject in the experiment. The respective outgroup preference filled the choice options to 100.

Stimulus type 1 represented stimuli where the ingroup preference for one of the two charities was the most obvious. For example, 95 ingroup members chose charity A while five outgroup members chose this option A. Option B was chosen by 13 ingroup members and 87 outgroup members. The ratio of ingroup/outgroup for option A (95/5) dominates the ingroup/outgroup ratio for option B (13/87); thus, the ingroup response is reflected in option A. The difference between the ingroup/outgroup ratio of charity A and charity B was then lower for stimulus type 2 (vs. stimulus type 1; e.g., 54/46 for A, 92/8 for B; B is the ingroup response here) and the lowest for stimulus type 3 (e.g., 54/46 for A, 13/87 B; A is the ingroup response here).

Thus, ingroup preference for one of the two charities was most evident in stimulus type 1, then 2 and then 3. Assuming that participants follow the ingroup, we assumed that it is more difficult to follow the ingroup when the ingroup/outgroup ratio (ingroup preference) between choices (A and B) is lower. Therefore, we expected ingroup conforming responses to be more frequent and faster in stimulus type 1 and consequently less in stimulus type 2. We expected the lowest proportion and the greatest difficulty in choosing the ingroup-compliant option in stimulus type 3.

### 2.4. Data Analysis

The data analysis was done in R (version 4.1.1; R Core Team, 2021). To estimate the DDM, we used the “rtdists” (version 0.11-2; Singmann et al., 2022) and “fddm” (version 0.4-0; Foster, 2022) package. We removed outlier trials for each participant separately, lying more than three interquartile ranges outside of the first and third quartiles of the log-transformed reaction time distribution (see Lerche and Voss, 2019). Furthermore, we removed trials with response times shorter than 200 ms (Schmitz and Voss, 2012). Overall, less than 1.26% of trials were removed. We used maximum likelihood (ML) with nlminb for parameter estimation. This algorithm provides stable parameter estimates for non-contaminated data, such as data without many outlier trials (Lerche et al., 2017).

#### 2.4.1. Drift Diffusion Model (DDM)

For the modeling approach, responses in line with the ingroup were linked to the lower boundary, and responses not in line with the ingroup were linked to the upper boundary. Within our analyses, we defined following the ingroup as choosing the charity that was most preferred by the ingroup (e.g., option A for stimulus type 1 and 3; option B for stimulus type 2; see Figure 2)

We tested our research questions by applying two different DDM groups. First, we expected the DDM variant, which allows for varying the drift parameter over the three different stimulus types, to provide a better Goodness of Fit than other DDM variants that do not allow for varying the drift. By allowing the drift to vary across the stimulus types, the model should capture hypothesized differences in the difficulty of the stimuli. The harder a task gets, the lower the drift should be. Therefore, it seemed reasonable to assume that one drift for all three stimulus types cannot represent this difference in difficulty appropriately. Second, we tested DDM variants to explain how subjects combine the ingroup/outgroup information in a joint evaluation scenario. Unlike the first variants, these variants explicitly consider the ingroup and outgroup information to model the decision by reformulating the drift (*v*) in a linear decomposition. A similar approach is known from Hierarchical Diffusion Models (e.g., Vandekerckhove et al., 2011; Wiecki et al., 2013). The decomposition of the drift has also been applied to non-hierarchical models, for example, when analyzing eye-tracking data (Krajbich et al., 2012). We expected the DDM variants that consider the ingroup and outgroup information (see Equation 1b) to outperform the DDM variants that only take into account the ingroup information (see Equation 1a).


(1a) $$\begin{array}{r} v=\beta_{0}+\beta_{1}*ingrInfo \end{array}$$

(1b) $$\begin{array}{r} v=\beta_{0}+\beta_{1}*ingrInfo+\beta_{2}*outgrInfo \end{array}$$

One way to use the trial information to inform the drift is to extract and weigh (β_1_) only the ingroup information (ingrInfo). However, in a joint evaluation scenario, both the ingroup and outgroup information could be extracted by decision-makers. So we implemented an additional weighting parameter β_2_ for the outgroup information (outgrInfo). In both cases, β_0_ represents the intercept, i.e., baseline level of evidence accumulation (compare with Trueblood et al., 2014).

#### 2.4.2. Trial Information

We tested five possible ways of extracting trial information for the in- and outgroup. First, the ratio of group members that chose option A and option B; For example, if 95 ingroup members chose A and 13 chose B, then the ratio A/B = 95/13 = 7.31 indicates that option A is 7.31 times more likely for ingroup members than option B. Second, the percentage of group members that chose option A; The percentage of previous ingroup donors for option A is A/(A+B) = 95/(95+13) = 0.88 indicates that 88% of the group members' choice was option A. Third, a more rudimentary abstraction is to set the extracted information into dichotomous information. In our case, we defined the item information to be 1 if the highest number (most of the donors) in the trial table belongs to the ingroup. We also tested models that use alternative-wise comparisons. Here we utilized direct (A-B) and relative A/(A-B) difference (see Dai and Busemeyer, 2014).

#### 2.4.3. Model Selection

The evaluation of Goodness-of-Fit was based on the Bayesian Information Criteria (BIC). We selected one model that had the lowest BIC value from each model group, indicating that this model fits the data, compared to all tested models, most accurately.

## 3. Results

Response times and choices were analyzed to test the effects of in- vs. outgroup information on individual donation decisions. Secondly, we used the Drift-Diffusion Model to understand how ingroup vs. outgroup information influenced charity preferences. For the analysis, we coded choices into “ingroup compliant” (Kandinsky) or “ingroup non-compliant”. Responses that point to the same choice option as the highest ingroup/outgroup ratio were classified as ingroup responses. Answers that do not refer to the highest ingroup/outgroup ratio were declared as ingroup non-compliant responses.

### 3.1. Behavioral Data

The behavioral data shows that 78% of the overall responses of our sample were ingroup compliant. The median response time for these responses was 697 ms (*M* = 863 ms; *SD* = 543 ms), while the median response time for ingroup non-compliant responses was 894 ms (*M* = 1,081 ms; *SD* = 667 ms). Comparing the three different stimulus types (see column *all participants* in Table 1), an increasing trend in ingroup compliant response times could be identified from stimulus type 1 to stimulus type 3. The choice frequency of ingroup-compliant responses showed a different pattern in contrast to the response time. Ingroup-compliant responses were least frequent on stimulus type 2, followed by stimulus type 3, while these responses were most frequent on stimulus type 1.

**Table 1:** Behavioral data—*all participants* vs. *ingroup driven* vs. *equality driven*.

**Stimulus**		**All participants**		**Ingroup driven**		**Equality driven**	
1	% Ingroup choices	0.86	(0.19)	0.95	(0.15)	0.63	(0.3)
	Ingroup RT	0.889	(0.39)	0.823	(0.361)	1.057	(0.53)
	Outgroup RT	1.008	(0.394)	1.01	(0.379)	1.005	(0.416)
2	% Ingroup choices	0.71	(0.27)	0.9	(0.24)	0.24	(0.37)
	Ingroup RT	0.893	(0.369)	0.869	(0.345)	0.96	(0.432)
	Outgroup RT	1.067	(0.559)	1.126	(0.627)	0.937	(0.424)
3	% Ingroup choices	0.76	(0.2)	0.74	(0.18)	0.83	(0.27)
	Ingroup RT	0.976	(0.474)	0.981	(0.468)	0.964	(0.492)
	Outgroup RT	1.123	(0.551)	1.161	(0.525)	1.03	(0.603)

*Mean responses and response times per stimuli type. RT in seconds. Standard Error in parentheses*.

To test whether the response times of the ingroup compliant responses differed between the three stimulus types, we conducted a repeated-measures ANOVA. Two participants were excluded from the analysis because they only gave ingroup non-compliant responses to one of the three stimulus types. The sphericity assumption was violated; thus, we applied a Greenhouse-Geisser correction. The stimulus types differed significantly from each other, *F*_(1.78, 64.24)_ = 4.91, $\eta_{G}^{2}$ = 0.020, *p* = 0.01. A *Post-hoc*-Tukey test showed that stimulus type 1 and 3 differed significantly from each other, *t*_(36)_ = 2.9, *p* = 0.02, while the others did not, all *ps* ≥ 0.17.

Pearson's product-moment correlation analyses were performed to investigate whether ingroup identification was related to (speed of) ingroup compliant responses. However, for stimuli 1, 2, and 3, the median response time, separated and aggregated over stimuli and responses, did not correlate significantly with group identity, all *ps* ≥ 0.07. Only the ingroup choices for stimulus type 3 correlated with ingroup identification, *r*_(37)_ = 0.44, *p* = 0.005, with higher ingroup identification being related to more ingroup compliant choices.

### 3.2. Modeling Approach of Prosocial Behavior

We created two large model groups in the diffusion approach (see Table 2). The first model group (30 models) was tested to determine which item information is used to form participants' decisions. We applied models only informed by the ingroup (β_1_) or by the ingroup and outgroup (β_1_ and β_2_) for all five different item information. We fixed for each combination the starting point *z* = 0.5, the drift variation *sv* = 0 or both parameter which resulted in 30 models.

**Table 2:** DDM group one.

**Model group 1**				
**Model**	**Par**	**Par fixed**	**Par vary**	**Item information**
1 (2)	*a, t*_0_, β_0_, β_1_, (β_2_), *sv*	*z*		*A*−*B*
3 (4)	*z, a, t*_0_, β_0_, β_1_, (β_2_)	*sv*		*A*−*B*
5 (6)	*a, t*_0_, β_0_, β_1_, (β_2_)	*z* + *sv*		*A*−*B*
7 (8)	*a, t*_0_, β_0_, β_1_, (β_2_), *sv*	*z*		*A*/(*A*−*B*)
9 (10)	*z, a, t*_0_, β_0_, β_1_, (β_2_)	*sv*		*A*/(*A*−*B*)
11 (12)	*a, t*_0_, β_0_, β_1_, (β_2_)	*z* + *sv*		*A*/(*A*−*B*)
13 (14)	*a, t*_0_, β_0_, β_1_, (β_2_), *sv*	*z*		*A*/*B*
15 (16)	*z, a, t*_0_, β_0_, β_1_, (β_2_)	*sv*		*A*/*B*
17 (18)	*a, t*_0_, β_0_, β_1_, (β_2_)	*z* + *sv*		*A*/*B*
19 (20)	*a, t*_0_, β_0_, β_1_, (β_2_), *sv*	*z*		*A*/(*A*+*B*)
21 (22)	*z, a, t*_0_, β_0_, β_1_, (β_2_)	*sv*		*A*/(*A*+*B*)
23 (24)	*a, t*_0_, β_0_, β_1_, (β_2_)	*z* + *sv*		*A*/(*A*+*B*)
25 (26)	*a, t*_0_, β_0_, β_1_, (β_2_), *sv*	*z*		1 or 0
27 (28)	*z, a, t*_0_, β_0_, β_1_, (β_2_)	*sv*		1 or 0
29 (30)	*a, t*_0_, β_0_, β_1_, (β_2_)	*z* + *sv*		1 or 0
**Model group 2**				
31, (32), [33]	*a*_*i*_, *t*_0_, *v*	*z*, (*sv*), [ *z* + *sv*]	*a*	−
34, (35), [36]	*a*, *t*_0_, *v*_*i*_	*z*, (*sv*), [ *z* + *sv*]	*v*	−
37, (38), [39]	*a*, *t*_0*i*_, *v*	*z*, (*sv*), [ *z* + *sv*]	*t*_0_	−
40, (41), [42]	*a*_*i*_, *t*_0_, *v*_*i*_	*z*, (*sv*), [ *z* + *sv*]	*a* + *v*	−
43, (44), [45]	*a*_*i*_, *t*_0*i*_, *v*	*z*, (*sv*), [ *z* + *sv*]	*a* + *t*_0_	−
46, (47), [48]	*a*, *t*_0*i*_, *v*_*i*_	*z*, (*sv*), [ *z* + *sv*]	*t*_0_ + *v*	−
49, (50), [51]	*a*_*i*_, *t*_0*i*_, *v*_*i*_	*z*, (*sv*), [ *z* + *sv*]	*a* + *t*_0_ + *v*	−

*Linear composition of drift v; parameter range for optimization: s = 1, β components: [−15;15], t_0_: [0;median(RT_ID_)], z [0;1]; sv [0;15], a [0;15], st0 = 0, sz = 0; fixed pars z =.5, sv = 0*.

We used a standard DDM with parameters varying across the three stimulus types in the second model group. For each specific model, we let either threshold *a*, drift rate *v*, non-decision time *t*_0_, or combinations of these parameters vary across stimulus conditions. For each combination we fixed either the starting point *z* = 0.5 (note that *z* is relative to the threshold), the drift variation *sv* = 0 or both of the parameters which resulted in 21 models. By utilizing this model group, we aimed to investigate which parameters can capture the different difficulties for the three types of stimuli.

**Figure 3:**
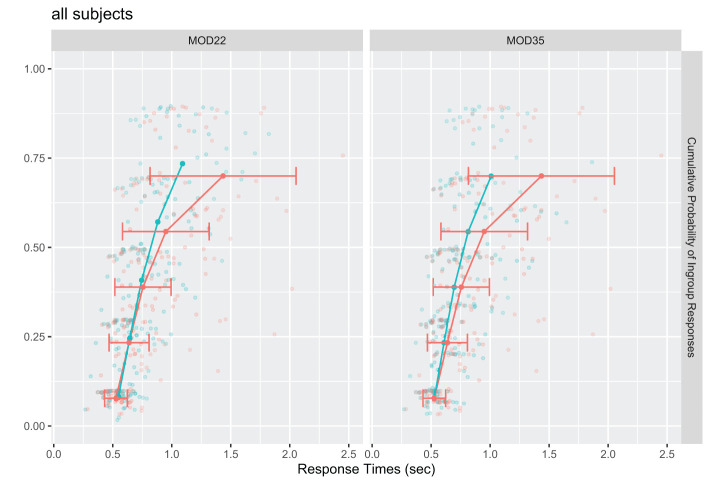
Cumulative Distribution Function Plot—predicted (red) vs. observed (blue) responses for model 22 and 35; quantiles per subject aggregated; only ingroup responses; lines represents 0.10th, 0.30th, 0.50th, 0.70th, 0.90th aggregated quantiles; dots are individual quantiles; standard deviation as error bar; red: observed data; blue: predicted data.

All models were fitted separately for each subject, and parameters were optimized by using the maximum likelihood algorithm. The best three mean model results for both model groups and aggregated across our sample can be found in Table 3. BIC values are highly similar for each group, indicating that each fits the data almost equally well. However, based on the best BIC, we chose model 22 and model 35. Further, both models showed a satisfying fit the 0.10th, 0.30th, 0.50th, and 0.70th quantiles (predicted/observed) overlap for ingroup responses (see Figure 3). However, the best fitting models underestimated observed response durations at the higher quantiles. For the 0.90ths quantile, a misfit produced. This is common in Diffusion Model approaches, especially for response times greater than 1 second. Extreme quantiles (i.e., 0.90 quantile) show a less satisfactory fit due to higher variability in response times (Aschenbrenner et al., 2016). The high variability in response times is visualized using the red error bar, which represents 1 for the respective quantiles. We would like to add that we did not exclude any participants and applied a conservative outlier handling.

**Table 3:** Mean model results of three best-fitting models for each model group and sample (*all participants* vs. *ingroup driven* vs. *equality driven*); TI, trial information used to inform β_1_ and β_2_; *z* is the relative bias; an empty cell in *z* or *sv* means, that the parameter is fixed to 0.5 and 0, respectively; an empty cell for *a*, or *t*_0_ means, that this parameter was fixed for all three stimuli types—otherwise the parameter was allowed to vary between the stimuli types.

**Mod**	**z**	**a**			***t*_0_**			**v**			***sv***	**BIC**	**TI**
								**β_0_**	**β_1_**	**β_2_**			
**Aggregated over all participants**													
22	0.48	1.99			0.32			1.92	−2.82	−1.11		271.86	A/(A+B)
10	0.49	1.99			0.32			−2.63	0.55	0.40		271.95	A/(A-B)
8		2.25			0.30			−3.44	0.67	0.52	0.8	272.75	A/(A-B)
		***a*** **1**	***a*** **2**	***a*** **3**	**t_0_1**	**t_0_2**	**t_0_3**	***v*** **1**	***v*** **2**	***v*** **3**			
35	0.48	2.01			0.32			−1.58	−0.88	−1.00		268.48	-
46		2.19			0.31	0.31	0.33	−2.08	−1.24	−1.41	0.81	268.95	-
34		2.27			0.30			−2.14	−1.26	−1.39	0.80	269.18	-
**Ingroup driven**													
22	0.47	1.99			0.32			2.22	−1.32	−3.27		188.29	A/(A+B)
10	0.47	1.99			0.32			−3.37	0.31	0.93		188.57	A/(A-B)
8		2.30			0.30			−4.47	0.43	1.12	0.87	189.27	A/(A-B)
		***a*** **1**	***a*** **2**	***a*** **3**	**t_0_1**	**t_0_2**	**t_0_3**	***v*** **1**	***v*** **2**	***v*** **3**			
35	0.47	2.01			0.32			−2.04	−1.60	−0.93		185.34	-
34		2.32			0.30			−2.80	−2.20	−1.44	0.87	185.99	-
46		2.24			0.30	0.31	0.34	−2.73	−2.16	−1.46	0.88	187.54	-
**Equality driven**													
12		1.98			0.30			−0.71	1.15	−0.94		484.12	A/(A-B)
10	0.52	1.99			0.31			−0.74	1.16	−0.95		484.19	A/(A-B)
24		1.98			0.30			1.24	−6.6	4.43		484.42	A/(A+B)
		***a*** **1**	***a*** **2**	***a*** **3**	**t_0_1**	**t_0_2**	**t_0_3**	***v*** **1**	***v*** **2**	***v*** **3**			
47	0.50	1.96			0.36			−0.38	0.98	−1.16		473.87	-
48		1.94			0.35			−0.37	0.97	−1.15	0.87	474.79	-
50	0.50	2.20	1.86	2.03	0.34	0.32	0.33	−0.44	0.94	−1.18	0.88	474.85	-

The basic parameter structure (see Table 3) is overlapping, indicating that the hypothesized item difficulty is mapped on the drift *v* parameter. For example, the best fitting models had *z* fixed to 0.5 or estimated the relative starting bias close to 0.5. The evidence threshold *a* did not vary across the stimuli types. Further, we found that the non-decision time component *t*_0_ was not varying over stimuli types for all but one model (model 46). For model group 2, the drift parameter varies across the three stimuli types. Altogether, it seems plausible that the item difficulty is mainly mapped on the drift *v* parameter of the DDM. Negative drift (*v*) and drift-components (β) for trial information A/(A+B) indicate evidence sampling toward the ingroup boundary (ingroup compliant response belongs to the lower boundary). The trial information A/(A-B) is vice versa, meaning that the negative drift and drift components indicate evidence sampling toward the non-ingroup boundary.

In model 22, the positive value for β_0_ is relevant to capture the evidence accumulation toward the non-ingroup compliant response since both weighting parameter β_1_ and β_2_ of the trial information A/(A+B) strongly tend to the ingroup compliant response (negative sign). Note that weighting parameters for models utilizing A/(A-B) trial information are interpreted in a way that negative values indicate evidence accumulation toward the non-ingroup compliant response.

In model 35, only the drift parameter *v* varied between the three stimulus types. The drift value was the largest for stimulus type 1, where the ingroup dominates the most. Contrary to our expectations, the smallest ingroup effect occurred for stimulus type 2 and not, as initially predicted, for stimulus type 3.

### 3.3. Group Identification and DDM Parameter

We computed Pearson's product-moment correlations to test whether individuals with stronger ingroup identification show stronger effects on the drift rate parameter. For model 35, the drift of stimulus type 3 correlated significantly with ingroup identification, *r*_(37)_ = -0.41, *p* = 0.009, whereas all other correlations were insignificant (*ps* > 0.09). Only the third stimulus type correlated with the cognitive process mainly responsible for responding in an ingroup compliant manner, such that stronger ingroup identification was related to faster and more ingroup compliant responses.

### 3.4. Classifying Participants as “Ingroup Driven” and “Equality Driven”

We performed further *post-hoc* analyses to investigate whether variations in model parameters can explain individual differences in participants' behaviors. Both DDM models showed unexpected results. Parameter estimates for model 22 (which uses in- and outgroup information) show that a median drift component β_2_ for the outgroup information of 0.02 (*M* = −1.11; *SD* = 5.91). However, the large standard deviation indicates that not all people were affected equally by the information provided.

The drifts for each stimuli type in model 35 further indicated that not all people were equally affected by other donors' group membership. We found the ingroup compliance effect across all stimulus types and in the individual stimuli. However, we were able to show through the modeling approach that the ingroup effect was stronger in stimulus type 1 than in stimulus type 3 and stimulus type 2 but weaker in stimulus type 2 than in stimulus type 3. We, therefore, concluded that some participants used a different decision strategy for stimulus type 2.

Participants' self-reported decision strategy suggested the possibility that some might have built their decisions on equality considerations, i.e., they chose the option that showed the smallest discrepancy between ingroup and outgroup. A reinspection of the experimental stimulus types revealed that, without this being initially intended, within stimulus type 2, participants could follow one of two strategies: follow the ingroup or choose equality. If they followed the ingroup, they chose the charity that presented the highest share from the ingroup independently of the preference of the outgroup. If they chose equality, participants chose the charity that minimized the difference in the share of the in- and outgroup (e.g., charity A: 92 share ingroup, 8 share outgroup; charity B: 54 share ingroup, 46 share outgroup). When being presented with stimulus type 3, participants could follow the ingroup and decide based on equality by choosing the same charity. In this stimulus type, one of the two options represents the charity most shared by the ingroup and the lowest difference in share between the ingroup and outgroup choices (e.g., charity A: 13 share ingroup, 87 share outgroup; charity B: 54 share ingroup, 46 share outgroup). Finally, stimulus type 1 presented a clear ingroup preference of the ingroup toward one of the two charities but did not present the possibility to choose equality since the difference between the share of the ingroup and outgroup in the two charities was large (e.g., charity A: 95 share ingroup, 5 share outgroup; charity B: 17 share ingroup, 83 share outgroup).

To investigate whether this possible alternative strategy would be reflected in the data, we split our sample into two strategy groups and reran our main analyses on an exploratory basis. Group assignment of participants was based on ingroup compliant behavior in stimulus type 2, as this stimulus forced participants to decide either to follow the ingroup or choose equality. Participants with 50% or less ingroup compliant decisions were assigned to the “equality driven” group (11 subjects). In contrast, those with more than 50% of ingroup compliant decisions were assigned to the “ingroup driven” group (28 subjects).

### 3.5. Rerunning Analyses

To rule out the possibility that the difference in behavior in the two subgroups merely resulted from a discrepancy of the group membership manipulation's effectiveness, we ran our manipulation check separately for both groups. A paired sample *t*-test indicated that our manipulation was successful for “ingroup driven” participants, as they showed significantly higher levels of empathy toward their ingroup (Kandinsky; *M* = 3.58, *SD* = 1.42) compared to the outgroup (Klee; *M* = 2.98, *SD* = 1.47), *t*_(27)_ = 3.90, *p* < 0.001, *d* = 0.73, 95% CI = [0.28, 0.92]. For the “equality driven” subjects, participants reported higher levels of empathy toward their ingroup (Kandinsky; *M* = 3.20, *SD* = 1.56) compared to the outgroup (Klee; *M* = 2.74, *SD* = 1.27). However, this difference was not significant, *t*_(10)_ = 2.18, *p* = 0.05, 95% CI = [−0.01, 0.92]. A sensitivity power analysis for the paired sample *t*-test suggested our sample size of 11 participants provided 80% power to detect a minimum effect size of *d* = 0.94, indicating that the sample size of the “equality driven” subgroup provided reasonable power only for detecting a considerably large effect.

#### 3.5.1. Ingroup Response Times for Ingroup and Equality Driven Participants

We reran the repeated-measures ANOVA for both participant groups to test whether “ingroup driven” and “equality driven” participants respond faster for ingroup compliant choices. One “ingroup driven” participant was excluded from the analysis because they only gave ingroup non-compliant responses to one of the three stimulus types. The sphericity assumption was violated; thus, we applied a Greenhouse-Geisser correction. The stimulus types differed significantly from each other, *F*_(1.21, 31.59)_ = 11.87, $\eta_{G}^{2}$ = 0.048, *p* < 0.001. A *Post-hoc*-Tukey test showed that stimulus type 1 and 3, *t*_(26)_ = 4.8, *p* < 0.001, and stimulus type 1 and 2, *t*_(26)_ = 4.4, *p* < 0.001, differed significantly, while stimulus type 2 and 3 did not, *p* = 0.15. For the equality “driven participants”, we did not find any significant differences for ingroup compliant response times for the stimulus types, *p* = 0.55 (see Table 1 for mean responses and response times aggregated across participants and aggregated for both groups).

#### 3.5.2. Modeling Results for Ingroup and Equality Driven Participants

By rerunning the analysis separately for both subgroups, the fit for ingroup-driven participants improved, while the fit for the equality-driven subjects became worse compared to the aggregated data (see Table 3). The best fitting model in the first model group, model 22, showed that outgroup information (mean [median] β_2_ = −3.27[−1.45]) and the ingroup information (β_1_ = −1.32 [−1.49]) for “ingroup driven” subjects accumulate toward a ingroup response. For the “equality driven” subjects, the best fitting model in group 1 shifted from model 22 to model 12. Responding ingroup compliant for “ingroup driven” subjects, model 35 showed that stimulus type 1 was easier than stimulus type 2, which in turn was easier than stimulus type 3.

Concerning ingroup identification, it showed that for “ingroup driven” participants, ingroup identification is negatively correlated with the drift for the third stimulus, *r*_(26)_ = −0.54, *p* = 0.003, indicating that participants were more ingroup compliant on this stimulus when their ingroup identification is higher.

## 4. Discussion

In this experiment, we aimed to investigate the effect of ingroup vs. outgroup information in a joint evaluation scenario by applying a DDM approach. Specifically, we argued that people would be more likely to follow the ingroup, i.e., charity options with a high ingroup ratio would be chosen faster and more often than charity options with a low ingroup ratio.

We tested our research questions by applying two different DDM groups. The first group investigated how participants used the presented ingroup/outgroup ratio in a joint evaluation scenario. The second model group investigated whether drift rate differences emerge when varying the ingroup/outgroup ratio. Model 22 for model group one and model 35 for model group two showed the best fit.

Results from model 22 confirmed our intuitions. We showed that it is easier for participants to follow the ingroup and that they were faster when they do so. As a result, participants were more likely to choose the charity most preferred by their ingroup. We also demonstrated on aggregated data that they were using the information provided about the ingroup preferences to follow the ingroup (i.e., a negative β_1_).

Within model 35, it showed that, unlike initially predicted, people most often and fastest decided ingroup compliant in stimulus type 1, followed by stimulus type 3, followed by stimulus type 2. This partially aligned with the initial assumption: people were fastest and most often decided according to their ingroup when the ingroup preference was most explicit and obvious. However, we observed a reverse pattern for stimuli 2 and 3, i.e., participants showed more and faster ingroup compliant decisions for stimuli with the least evident ingroup preference compared to stimuli where ingroup preference was less obvious but still clearly evident.

When examining the stimulus types more closely, it became apparent that participants might have used two different strategies in their decision process, as some decision options reflected following the ingroup. In contrast, other options allowed decisions based on fairness and equality considerations. While stimulus type 1 lacked an option for equality, the nature of stimulus type 2 forced participants to choose between an option that reflected following the ingroup or following equality. For stimulus type 3, following the ingroup and following equality were reflected in the same decision option. This might explain why more and faster ingroup compliant decisions were observed in stimulus type 3 compared to stimulus type 2, as here ingroup compliant decisions did not conflict with the alternative decision strategy of equality that participants might have used.

Based on participants' self-reported strategy and participants' decisions for ingroup compliance or equality in stimulus type 2, we sorted participants by type of strategy that might be reflected in their decisions into an “ingroup driven”, and “equality driven” group and reran our analyses separately for these groups. When we reran the model group 1 for the “ingroup driven”, the same models as for the aggregated data showed a superior fit. Further, an increase in the model fit could be found. The “ingroup driven” also used the outgroup and ingroup preferences to follow the ingroup (i.e., negative drift component for trial information A/(A+B)). The higher the outgroup preference for one charity, the more likely they chose the other, ingroup preferred charity. We can speculate that people who blindly conform to their ingroup use the ingroup and outgroup information to maximize their conformity. For the “equality driven” participants, slightly different best-fitting models were found, although these models used the same information type as the best fitting models for the “ingroup driven”. “Equality driven” participants used the ingroup preferences to decide in favor of the ingroup (i.e., negative drift component see model 24) and the outgroup preferences to follow the outgroup (i.e., positive drift component see model 24). In this case, we can speculate that they were less biased toward the ingroup, therefore using more equally the information provided.

When we reran model group 2, we showed that for “ingroup driven” participants, the best-fitting models were also the best fitting models for the aggregated data. “Ingroup driven” participants displayed highly and particularly fast ingroup compliant decisions. Moreover, “ingroup driven” participants behaved in a way that matched our initial prediction: they showed the most and fastest ingroup compliant decisions for stimulus type 1, followed by stimulus type 2, followed by stimulus type 3. The “equality driven” participants, in contrast, did not show the same level of ingroup compliance in their decisions. For this group, the highest and fastest ingroup compliant decisions emerged for stimulus type 3, where following the ingroup and following equality was reflected within the same decision option, followed by stimulus type 1. In stimulus type 2, where participants were forced to choose between following the ingroup or equality, “equality driven” participants more often and faster decided on the better-fitted equality option rather than an ingroup compliant approach. As for the ingroup orientated participants, the stimulus difficulty seemed to be mapped on the drift rate.

Based on these results, we might speculate that participants indeed used two different strategies when deciding which charity they should donate to: While most people followed the ingroup in all of the decision scenarios, there was also a smaller group of participants that seemingly strived for equality when circumstances allowed it. However, none of the tested models in the present study captured the presumed equality-oriented behavior well for these equality-oriented participants. Future research could address such equality-based models systematically.

Overall, our results align with research showing that people tend to favor their ingroups and orient toward other ingroup members. Information on ingroup members has been found to trigger greater in-depth processing reflected in neuronal activity (Bavel et al., 2008). People also show better performance in remembering information somehow associated with an ingroup, even if this association is incidental rather than substantial (Jeon et al., 2021). On a cognitive level, this suggests that ingroup information automatically attracts attention as it is considered as more relevant to the self. Besides conformity effects arising from the desire to be socially accepted by other group members, in our context of minimal groups, it seems reasonable that people used ingroup members' most favored decision as a heuristic for how to behave, for what is “the right thing to do.” Indeed, research on morality judgments shows that group membership plays an essential role when people use a “what is common is good” heuristic. Whereas commonality of behavior among ingroup members is used as an indicator for the behavior's morality, its commonality among outgroups is rather irrelevant or weakly related to morality judgments (Goldring and Heiphetz, 2020). While former research has already established such an ingroup sensitivity effect in donation decisions when either information on in- or outgroup members' behavior is presented (Hysenbelli et al., 2013), we were able to extend these findings to situations where people were simultaneously confronted with in- and outgroup behavior.

At the same time, our results are also consistent with literature pointing out that the extent of intergroup bias might be dependent on interindividual differences. Specifically, value and social orientations have been found to moderate the strength of intergroup bias (Hewstone et al., 2002). For example, humanitarian and egalitarian values are related to lower prejudice and more positive intergroup attitudes across different types of outgroups (Biernat et al., 1996; Biernat and Vescio, 2005). For individuals personally motivated to avoid prejudice, automatic activation of egalitarian goals even alleviates implicit forms of negative outgroup bias (Johns et al., 2008). In fact, it has been argued that individuals' endorsement of anti-egalitarianism or situations where some social groups dominate others is a stable trait called Social Dominance Orientation (SDO) that predicts negative intergroup attitudes (Sidanius and Pratto, 1999). Preferential allocation to the ingroup has also been found to be correlated with SDO in a minimal group setting (Amiot and Bourhis, 2005). Similarly, social value orientation (SVO) has been shown to moderate ingroup favoritism in a conflict setting (De Dreu, 2010). Furthermore, individuals with a prosocial value orientation (vs. a pro-self-value orientation) invest more effort and spend more time on information search in an outgroup decision setting (Rahal et al., 2020). This suggests that individuals with fairness concerns are more likely to pay attention to outgroup information when making a prosocial decision – a conclusion also supported by the present research.

These findings, however, typically refer to intergroup bias reflected in attitudes toward or treatment of outgroup compared to ingroup members. Based on the results of our study, one might speculate that traits such as SVO also influence intergroup bias when it comes to decision formation with outcomes unrelated to the in- and outgroup (i.e., the target of donation was never an in- or outgroup member but rather a third party with no group membership stated). To be able to validate this presumption, future research might thus examine whether explicit measures of social value orientations are related to participants' ingroup conformity when information on in- and outgroup behavior is presented.

In the aggregated data analysis for model 51, we found a correlation between the drift parameter and group identity for stimulus type 3, indicating that the stronger people identified with their ingroup, the more likely they decided to choose it. However, we did not find a significant correlation between group identification and the drift parameters of stimulus types 1 and 2. One potential reason we find this pattern of results could be that, in stimulus type 3, compared to the other stimuli, the ingroup has only a slight preference for one charity over the other [e.g., Charity A: 55 (ingroup)/45 (outgroup), Charity B: 13 (ingroup)/87 (outgroup)]. In other words, out of all the stimuli, stimulus type 3 is the most ambiguous with regard to ingroup preference. Therefore, we can speculate that people with higher ingroup identification were more likely to follow the ingroup when making a decision based on highly ambiguous group preference information.

We also investigated the correlation between group identification and drift parameters for each of the two groups (i.e., “ingroup driven” and “equality driven”). For participants classified as “ingroup driven”, we find the exact same pattern that we found for the aggregated data, i.e., a significant correlation between drift parameter and group identification for stimulus type 3, but none for stimulus type 1 and 2. However, for “equality driven” participants, we found no significant correlation between group identification and drift parameters (participants' likelihood to follow the ingroup). Thus, the pattern we found for the aggregated data was mainly due to the behavior of the “ingroup driven” participants and not the “equality driven” participants. “Ingroup driven” participants with higher group identification were more likely to follow the ingroup even in an ambiguous context. This lends support to our behavioral classification of participants as “ingroup driven” and “equality driven”.

In general, higher ingroup identification can be but is not necessarily connected to greater intergroup bias (Hewstone et al., 2002; Dovidio and Gaertner, 2010). In our study on the effect of intergroup social influence on individual decision making, ingroup identification only played a role for stimulus type 3, which was the only stimulus type where the number of outgroup donors outweighed the number of ingroup donors. Although being speculative at this point, this dominance of the outgroup might have been perceived as an implicit threat to the ingroup's power and sovereignty. For natural groups (i.e., immigrants), it has been found that outgroup size is positively related to higher levels of perceived intergroup threat and discriminatory attitudes (Schlueter and Scheepers, 2010). Additionally, intergroup bias is known to increase under perceptions of group threat, and such effects tend to be stronger for those with higher levels of ingroup identification (e.g., Smurda et al., 2006; Rios et al., 2018). Highly identified individuals also express enhanced conformity to ingroup specific values when they consider their ingroup as being threatened (Jetten et al., 2002; Morrison and Ybarra, 2009), and they show more loyalty to a low-status ingroup when being given the possibility to move to a higher status outgroup (Ellemers et al., 1997). Thus, if participants in our experiment indeed regarded the outgroup dominance as a kind of threat, it would seem reasonable that the high identifiers among “ingroup driven” participants were even more likely to stand in line with the ingroup when making their decision for this stimulus type.

There are limitations we must take into account when interpreting our results. One limitation is that this study was done in an artificial, laboratory online experiment, making it unclear how these findings would translate to real-world scenarios. While this allows us to identify basic psychological processes under high internal validity, future studies might benefit from building on our findings in more naturalistic settings. For example, we gave participants a contrived choice with limited information, which is not typical in most donation settings, and used minimal instead of natural groups. Although minimal groups have several advantages, such as a lack of confounding factors that arise from known stereotypes, the external validity of this paradigm is low. Thus, it is unclear whether the current findings regarding the classification of participants' into “equality driven” and “ingroup driven” individuals would hold for real-world groups. “Equality driven” individuals might not be motivated by fairness if the outgroup charity supported a cause that was particularly abhorrent to them (e.g., a racist organization). Future research that attempts to replicate our *post-hoc* analysis should also investigate under what group contexts these two different strategies emerge. Additionally, the focus should shift from models that describe aggregated data to models that best fit single strategies, particularly if these strategies can be replicated successfully in future studies. Further, our experimental design did not allow us to capture all possible kinds of ingroup vs. outgroup proportions (e.g., cases where options were weakly preferred by more outgroup than ingroup members). Thus, future research might benefit from adding more variety within the stimulus types.

Our analytic strategy also has a few limitations. The DDM approach assumes a single-stage process. Although our results suggest that participants maintain their strategy across all stimuli (indicating a single-stage process), we cannot rule out the possibility that the initially chosen strategy is replaced by another strategy in the same decision-making process (multi-stage process). Furthermore, the best-fitting parameter (point estimate) is determined using maximum likelihood estimation. By applying Bayesian estimation, one can use the highest density interval (HDI) for each parameter (e.g., the drift *v*) within participants to test for intra-individual differences between stimulus types. A plausible assumption might be that the HDI should be small for easy choices, as opposed to difficult choices.

Future research should focus on these parameter deviations as an indicator of the strength of commitment to a strategy. For example, lower drift parameter deviation may indicate greater certainty in participants' strategy choices. While keeping these limitations in mind, the present research provides first valuable insights into the cognitive process underlying donation decisions when information on ingroup and outgroup members' behavior is presented simultaneously. Specifically, the DDM approach revealed two types of donors that process in vs. outgroup information differently. Although people generally tended to follow the ingroup through their decisions, there was also a group trying to minimize differences between the in- and outgroup, therefore trying to be as unbiased as possible toward their ingroup when being presented with the behavior of both groups at the same time. Recognizing this may have important implications when using a social norm and social identity approach for donor acquisition and can help charities and other fund raising organizations in designing tailored and effective campaigns for their causes and target groups.

## Data Availability Statement

The datasets presented in this study can be found in online repositories. The names of the repository/repositories and accession number(s) can be found below: https://osf.io/6kv3b/?view_only=dfe7e6a6801f410a84daa2f21b9fbd9c.

## Ethics Statement

The studies involving human participants were reviewed and approved by Ethics at the University of Klagenfurt (ER-AAU). The patients/participants provided their written informed consent to participate in this study.

## Author Contributions

LR: conceptualization, formal analysis, visualization, writing, reviewing, and editing. LM, MV, and AK: conceptualization, writing, reviewing, and editing. All authors contributed to the article and approved the submitted version.

## Conflict of Interest

The authors declare that the research was conducted in the absence of any commercial or financial relationships that could be construed as a potential conflict of interest.

## Publisher's Note

All claims expressed in this article are solely those of the authors and do not necessarily represent those of their affiliated organizations, or those of the publisher, the editors and the reviewers. Any product that may be evaluated in this article, or claim that may be made by its manufacturer, is not guaranteed or endorsed by the publisher.

## References

[B1] Aberson C. L. Healy M. Romero V. (2000). Ingroup bias and self-esteem: a meta-analysis. Pers. Soc. Psychol. Rev. 4, 157–173. 10.1207/S15327957PSPR0402_04

[B2] Abrams D. Hogg M. A. (1990). Social identification, self-categorization and social influence. Eur. Rev. Soc. Psychol. 1, 195–228. 10.1080/1479277910840186232931718

[B3] Amiot C. E. Bourhis R. Y. (2005). Ideological beliefs as determinants of discrimination in positive and negative outcome distributions. Eur. J. Soc. Psychol. 35, 581–598. 10.1002/ejsp.238

[B4] Aschenbrenner A. J. Balota D. A. Gordon B. A. Ratcliff R. Morris J. C. (2016). A diffusion model analysis of episodic recognition in preclinical individuals with a family history for Alzheimer's disease: the adult children study. Neuropsychology 30, 225–238. 10.1037/neu0000222 26192539 PMC4720576

[B5] Batson C. D. Polycarpou M. P. Harmon-Jones E. Imhoff H. J. Mitchener E. C. Bednar L. L.. (1997). Empathy and attitudes: can feeling for a member of a stigmatized group improve feelings toward the group? J. Pers. Soc. Psychol. 72, 105–118. 10.1037/0022-3514.72.1.105 9008376

[B6] Bavel J. J. V. Packer D. J. Cunningham W. A. (2008). The neural substrates of in-group bias: a functional magnetic resonance imaging investigation. Psychol. Sci. 19, 1131–1139. 10.1111/j.1467-9280.2008.02214.x 19076485

[B7] Bernheim B. D. (1994). A theory of conformity. J. Polit. Econ. 102, 841–877. 10.1086/261957

[B8] Biernat M. Vescio T. K. (2005). “Values and prejudice,” in Social Psychology of Prejudice: Historical and Contemporary Issues, eds C. S. Crandall and M. Schaller (Lawrence, KS: Lewinian Press), 191–216.

[B9] Biernat M. Vescio T. K. Theno S. A. (1996). Violating American values: a “value congruence” approach to understanding outgroup attitudes. J. Exp. Soc. Psychol. 32, 387–410. 10.1006/jesp.1996.0018

[B10] Bornstein G. Crum L. Wittenbraker J. Harring K. Insko C. A. Thibaut J. (1983). On the measurement of social orientations in the minimal group paradigm. Eur. J. Soc. Psychol.Q 13, 321–350. 10.1002/ejsp.2420130402

[B11] Caviola L. Faulmüller N. Everett J. A. Savulescu J. Kahane G. (2014). The evaluability bias in charitable giving: saving administration costs or saving lives? Judgment Decis. Mak. 9, 303–316. 10.13140/2.1.1028.9287 25279024 PMC4179876

[B12] Cialdini R. B. Goldstein N. J. (2004). Social influence: compliance and conformity. Annu. Rev. Psychol. 55, 591–621. 10.1146/annurev.psych.55.090902.142015 14744228

[B13] Dai J. Busemeyer J. R. (2014). A probabilistic, dynamic, and attribute-wise model of intertemporal choice. Exp. Psychol. 143, 1489–1514. 10.1037/a0035976 24635188 PMC4115005

[B14] De Dreu C. K. (2010). Social value orientation moderates ingroup love but not outgroup hate in competitive intergroup conflict. Group Process. Intergroup Relat. 13, 701–713. 10.1177/1368430210377332

[B15] Doosje B. Ellemers N. Spears R. (1995). Perceived intragroup variability as a function of group status and identification. J. Exp. Soc. Psychol. 31, 410–436. 10.1006/jesp.1995.1018

[B16] Dovidio J. F. Gaertner S. L. (2010). “Intergroup bias,” in Handbook of Social Psychology, eds S. T. Fiske, D. T. Gilbert, and G. Lindzey (New York, NY: Wiley), 1084–1121. 10.1002/9780470561119.socpsy002029

[B17] Ellemers N. Spears R. Doosje B. (1997). Sticking together or falling apart: in-group identification as a psychological determinant of group commitment versus individual mobility. J. Pers. Soc. Psychol. 72, 617–626. 10.1037/0022-3514.72.3.617

[B18] Festinger L. (1954). A theory of social comparison processes. Hum. Relat. 7, 117–140. 10.1177/001872675400700202

[B19] Foster K. B. (2022). fddm: Fast Implementation of the Diffusion Decision Model. R package version 0.5-1. Available online at: https://CRAN.R-project.org/package=fddm

[B20] Goldring M. R. Heiphetz L. (2020). Sensitivity to ingroup and outgroup norms in the association between commonality and morality. J. Exp. Soc. Psychol. 91, 104025. 10.1016/j.jesp.2020.104025

[B21] Goldstein N. J. Cialdini R. B. Griskevicius V. (2008). A room with a viewpoint: using social norms to motivate environmental conservation in hotels. J. Cons. Res. 35, 472–482. 10.1086/586910

[B22] Henninger F. Shevchenko Y. Mertens U. K. Kieslich P. J. Hilbig B. E. (2021). lab.js: a free, open, online study builder. Behav. Res. Methods. 54, 556–573. 10.3758/s13428-019-01283-5 34322854 PMC9046347

[B23] Hewstone M. Rubin M. Willis H. (2002). Intergroup bias. Annu. Rev. Psychol. 53, 575–604. 10.1146/annurev.psych.53.100901.135109 11752497

[B24] Hysenbelli D. Rubaltelli E. Rumiati R. (2013). Others' opinions count, but not all of them: anchoring to ingroup versus outgroup members' behavior in charitable giving. Judgment Decis. Mak. 8, 678–690.

[B25] James T. K. Zagefka H. (2017). The effects of group memberships of victims and perpetrators in humanly caused disasters on charitable donations to victims. J. Appl. Soc. Psychol. 47, 446–458. 10.1111/jasp.12452

[B26] Jeon Y. A. Banquer A. M. Navangul A. S. Kim K. (2021). Social group membership and an incidental ingroup-memory advantage. Q. J. Exp. Psychol. 74, 166–178. 10.1177/1747021820948721 32705946

[B27] Jetten J. Postmes T. McAuliffe B. J. (2002). ‘We're all individuals': group norms of individualism and collectivism, levels of identification and identity threat. Eur. J. Soc. Psychol. 32, 189–207. 10.1002/ejsp.65

[B28] Johns M. Cullum J. Smith T. Freng S. (2008). Internal motivation to respond without prejudice and automatic egalitarian goal activation. J. Exp. Soc. Psychol. 44, 1514–1519. 10.1016/j.jesp.2008.07.003

[B29] Knippenberg D. V. Wilke H. (1992). Prototypicality of arguments and conformity to ingroup norms. Eur. J. Soc. Psychol. 22, 141–155. 10.1002/ejsp.2420220204

[B30] Kogut T. Ritov I. (2005). The “identified victim” effect: an identified group, or just a single individual? J. Behav. Decis. Mak. 18, 157–167. 10.1002/bdm.492

[B31] Krajbich I. Lu D. Camerer C. Rangel A. (2012). The attentional drift-diffusion model extends to simple purchasing decisions. Front. Psychol. 3, 193. 10.3389/fpsyg.2012.00193 22707945 PMC3374478

[B32] Lede E. Meleady R. Seger C. R. (2019). Optimizing the influence of social norms interventions: APPLYING social identity insights to motivate residential water conservation. J. Environ. Psychol. 62, 105–114. 10.1016/j.jenvp.2019.02.011

[B33] Lee S. Feeley T. H. (2016). The identifiable victim effect: a meta-analytic review. Soc. Influence 11, 199–215. 10.1080/15534510.2016.1216891

[B34] Lerche V. Voss A. (2019). Experimental validation of the diffusion model based on a slow response time paradigm. Psychol. Res. 83, 1194–1209. 10.1007/s00426-017-0945-8 29224184

[B35] Lerche V. Voss A. Nagler M. (2017). How many trials are required for parameter estimation in diffusion modeling? A comparison of different optimization criteria. Behav. Res. Methods 49, 513–537. 10.3758/s13428-016-0740-2 27287445

[B36] Minguez A. Sese F. J. (2021). Understanding the effectiveness of social influence appeals in charitable giving: the roles of affinity with the cause, and past giving behavior. J. Market. Theory Pract. 29, 375–386. 10.1080/10696679.2020.1859335

[B37] Morrison K. R. Ybarra O. (2009). Symbolic threat and social dominance among liberals and conservatives: SDO reflects conformity to political values. Eur. J. Soc. Psychol. 39, 1039–1052. 10.1002/ejsp.606

[B38] Palan S. Schitter C. (2018). Prolific.ac–A subject pool for online experiments. J. Behav. Exp. Fin. 17, 22–27. 10.1016/j.jbef.2017.12.004

[B39] Payne J. W. Payne J. W. Bettman J. R. Johnson E. J. (1993). The Adaptive Decision Maker. Cambridge University Press.

[B40] R Core Team (2021). R: A Language and Environment for Statistical Computing. Vienna: R Foundation for Statistical Computing.

[B41] Rahal R.-M. Fiedler S. De Dreu C. K. (2020). Prosocial preferences condition decision effort and ingroup biased generosity in intergroup decision-making. Sci. Rep. 10, 1–11. 10.1038/s41598-020-64592-2 32576839 PMC7311554

[B42] Ratcliff R. (1978). A theory of memory retrieval. Psychol. Rev. 85, 59–108. 10.1037/0033-295X.85.2.59

[B43] Ratcliff R. McKoon G. (2008). The diffusion decision model: theory and data for two-choice decision tasks. Neural Comput. 20, 873–922. 10.1162/neco.2008.12-06-420 18085991 PMC2474742

[B44] Ratcliff R. Rouder J. N. (1998). Modeling response times for two-choice decisions. Psychol. Sci. 9, 347–356. 10.1111/1467-9280.00067

[B45] Ratcliff R. Smith P. L. Brown S. D. McKoon G. (2016). Diffusion decision model: current issues and history. Trends Cogn. Sci. 20, 260–281. 10.1016/j.tics.2016.01.007 26952739 PMC4928591

[B46] Rios K. Sosa N. Osborn H. (2018). An experimental approach to intergroup threat theory: Manipulations, moderators, and consequences of realistic vs. symbolic threat. Eur. Rev. Soc. Psychol. 29, 212–255. 10.1080/10463283.2018.1537049

[B47] Schlueter E. Scheepers P. (2010). The relationship between outgroup size and anti-outgroup attitudes: a theoretical synthesis and empirical test of group threat- and intergroup contact theory. Soc. Sci. Res. 39, 285–295. 10.1016/j.ssresearch.2009.07.006

[B48] Schmitz F. Voss A. (2012). Decomposing task-switching costs with the diffusion model. J. Exp. Psychol. Hum. Percept. Perform. 38, 222. 10.1037/a0026003 22060144

[B49] Schultz P. W. Nolan J. M. Cialdini R. B. Goldstein N. J. Griskevicius V. (2007). The constructive, destructive, and reconstructive power of social norms. Psychol. Sci. 18, 429–434. 10.1111/j.1467-9280.2007.01917.x 17576283

[B50] Sidanius J. Pratto F. (1999). Social Dominance: An Intergroup Theory of Social Hierarchy and Oppression. Cambridge: Cambridge University Press. 10.1017/CBO9781139175043

[B51] Singmann H. Brown S. Gretton M. Heathcote A. (2022). rtdists: Response Time Distributions. R package version 0.11-5. Available online at: https://CRAN.R-project.org/package=rtdists

[B52] Small D. A. Loewenstein G. Slovic P. (2007). Sympathy and callousness: the impact of deliberative thought on donations to identifiable and statistical victims. Organ. Behav. Hum. Decis. Process. 102, 143–153. 10.1016/j.obhdp.2006.01.005

[B53] Smurda J. D. Wittig M. A. Gokalp G. (2006). Effects of threat to a valued social identity on implicit self-esteem and discrimination. Group Process. Intergroup Relat. 9, 181–197. 10.1177/1368430206062076

[B54] Tajfel H. (1970). Experiments in intergroup discrimination. Sci. Am. 223, 96–103. 10.1038/scientificamerican1170-96 5482577

[B55] Tajfel H. Turner J. C. (1979). “An integrative theory of intergroup conflict,” in The social psychology of intergroup relations, eds W. G. Austin and S. Worchel (Monterey, CA: Brooks/Cole), 33–47.

[B56] Trueblood J. S. (2012). Multialternative context effects obtained using an inference task. Psychon. Bull. Rev. 19, 962–968. 10.3758/s13423-012-0288-9 22736437

[B57] Trueblood J. S. Brown S. D. Heathcote A. (2014). The multiattribute linear ballistic accumulator model of context effects in multialternative choice. Psychol. Rev. 121, 179. 10.1037/a0036137 24730597

[B58] Turner J. C. (1999). Some current issues in research on social identity and self-categorization theories. Soc. Identity Context Commitment Content 3, 6–34.8510049

[B59] Van Lange P. A. Bekkers R. Schuyt T. N. Vugt M. V. (2007). From games to giving: social value orientation predicts donations to noble causes. Basic Appl. Soc. Psychol. 29, 375–384. 10.1080/01973530701665223

[B60] van Teunenbroek C. Bekkers R. Beersma B. (2020). Look to others before you leap: a systematic literature review of social information effects on donation amounts. Nonprofit Volunt. Sec. Q. 49, 53–73. 10.1177/0899764019869537

[B61] Vandekerckhove J. Tuerlinckx F. Lee M. D. (2011). Hierarchical diffusion models for two-choice response times. Psychol. Methods 16, 44. 10.1037/a0021765 21299302

[B62] Voss A. Nagler M. Lerche V. (2013). Diffusion models in experimental psychology. Exp. Psychol. 60, 1–18. 10.1027/1618-3169/a000218 23895923

[B63] Voss A. Rothermund K. Voss J. (2004). Interpreting the parameters of the diffusion model: an empirical validation. Mem. Cogn. 32, 1206–1220. 10.3758/BF03196893 15813501

[B64] Wagenmakers E.-J. (2009). Methodological and empirical developments for the ratcliff diffusion model of response times and accuracy. Eur. J. Cogn. Psychol. 21, 641–671. 10.1080/09541440802205067

[B65] Wiecki T. V. Sofer I. Frank M. J. (2013). HDDM: hierarchical Bayesian estimation of the drift-diffusion model in Python. Front. Neuroinform. 7, 14. 10.3389/fninf.2013.00014 23935581 PMC3731670

